# Efficient Selective Capture of Carbon Dioxide from Nitrogen and Methane Using a Metal-Organic Framework-Based Nanotrap

**DOI:** 10.3390/molecules28237908

**Published:** 2023-12-02

**Authors:** Junjie Peng, Chengmin Fu, Jiqin Zhong, Bin Ye, Jing Xiao, Chongxiong Duan, Daofei Lv

**Affiliations:** 1 School of Environmental and Chemical Engineering, Foshan University, Foshan 528000, China; cepengjunjie@fosu.edu.cn (J.P.); callinia@foxmail.com (C.F.); 2 GAC R&D Center, Guangzhou Automobile Group Co., Ltd., 668 Jinshan Road East, Panyu District, Guangzhou 511434, China; jackingzhong@163.com; 3 Appraisal Center for Environment and Engineering, Ministry of Ecology and Environment, Beijing 100012, China; yebin@acee.org.cn; 4 School of Chemistry and Chemical Engineering, South China University of Technology, Guangzhou 510640, China; cejingxiao@scut.edu.cn; 5 School of Materials Science and Hydrogen Engineering, Foshan University, Foshan 528000, China; cechxduan@fosu.edu.cn

**Keywords:** metal-organic framework, CO_2_ capture, nanotrap, gas separation

## Abstract

Selective carbon capture from exhaust gas and biogas, which mainly involves the separation of CO_2_/N_2_ and CO_2_/CH_4_ mixtures, is of paramount importance for environmental and industrial requirements. Herein, we propose an interesting metal-organic framework-based nanotrap, namely ZnAtzCO_3_ (Atz^−^ = 3-amino-1,2,4-triazolate, CO_3_^2−^ = carbonate), with a favorable ultramicroporous structure and electrostatic interactions that facilitate efficient capture of CO_2_. The structural composition and stability were verified by FTIR, TGA, and PXRD techniques. Particularly, ZnAtzCO_3_ demonstrated high CO_2_ capacity in a wide range of pressures, with values of 44.8 cm^3^/g at the typical CO_2_ fraction of the flue gas (15 kPa) and 56.0 cm^3^/g at the CO_2_ fraction of the biogas (50 kPa). Moreover, ultrahigh selectivities over CO_2_/N_2_ (15:85, *v:v*) and CO_2_/CH_4_ (50:50, *v:v*) of 3538 and 151 were achieved, respectively. Molecular simulations suggest that the carbon atom of CO_2_ can form strong electrostatic C^δ+^···^δ−^O-C interactions with four oxygen atoms in the carbonate ligands, while the oxygen atom of CO_2_ can interact with the hydrogen atoms in the triazolate ligands through O^δ−^···^δ+^H-C interactions, which makes ZnAtzCO_3_ an optimal nanotrap for CO_2_ fixation. Furthermore, breakthrough experiments confirmed excellent real-world separation toward CO_2_/N_2_ and CO_2_/CH_4_ mixtures on ZnAtzCO_3_, demonstrating its great potential for selective CO_2_ capture.

## 1. Introduction

Global warming, one of the biggest global issues, is causing various long-term disastrous environmental effects, including abnormal climate patterns, rising sea levels, accelerated extinction of species, and shifts in agricultural patterns, which pose severe threats to the survival and development of humanity [1,2,3,4]. As the main source of greenhouse gases, carbon dioxide (CO_2_), with a large annual emission (e.g., 36.8 Gt in 2022), contributes to 69.4% of the anthropogenic greenhouse gases [5,6]. Hence, CO_2_ capture and utilization is of utmost importance to curb global warming [7]. In particular, electricity generation from the combustion of fossil fuels, resulting in exhaust gas composed mainly of CO_2_ and nitrogen (N_2_), is the primary source of anthropogenic CO_2_ emissions [8,9]. Additionally, biogas, acknowledged as “renewable natural gas”, is a green fuel with the efficient component of methane (CH_4_) [10]. Nevertheless, biogas contains a considerable amount of CO_2_ that could significantly decrease the calorific value and lead to severe erosion in the equipment. Therefore, efficient selective capture of CO_2_ from N_2_ and CH_4_ is of paramount significance for environmental protection and biogas upgrading.

Benefiting from a plausible gentle operation condition and reduced energy consumption for regeneration, adsorptive separation of CO_2_ has been recognized as a promising alternative among the current CO_2_ capture techniques [11,12,13,14]. Fundamentally, an ideal adsorbent with high capacity and selectivity for the target molecule is the key to achieving satisfactory separation performance [15,16]. As a class of innovative porous adsorbents, metal-organic frameworks (MOFs) are showing extraordinary versatility in pore tunability and chemical functionalities [17]. Hence, adsorptive separation based on MOFs has been explored in miscellaneous separation circumstances, including carbon capture [8,9,11,18,19,20]. In general, there are several strategies that can enhance the CO_2_ capture performance on MOF materials, including (i) incorporation of functionalities (such as open metal sites, Lewis basic sites, and polar functional groups) into the frameworks, (ii) utilization of ultramicropores to maximize the confinement effect, (iii) use of kinetic difference and even the molecular sieving effect, (iv) introduction of structural flexibility, (v) the combination thereof [16,21,22]. Specifically, MOFs constructed by triazolate linkers represent one subclass of MOFs that are comprised of excellent CO_2_ capture performance, affordable costs, and good stability under humid conditions [23,24]. For example, by introducing amino groups into the triazolate linkers of the prototype MOF ZnF(TZ), the resultant ZnF(daTZ) demonstrates an appreciable volumetric CO_2_ uptake of 75 cm^3^/cm^3^ and high CO_2_/N_2_ equilibrium selectivity of 120 based on the ideal adsorbed solution theory (IAST) as well as excellent CO_2_/H_2_O kinetic selectivity of 70 [25]. This remarkable separation performance was probably originated from the main adsorptive site at the channel center for CO_2_ to afford electrostatic interactions with the amino groups, while H_2_O was more likely to locate at the channel corner, according to GCMC simulations. Built from dual ligands of oxalate and 1,2,4-triazolate, zinc-based Calgary Framework 20 (CALF-20) exhibits a high CO_2_ capacity of 87.36 cm^3^/g at atmospheric conditions, excellent selectivities over N_2_ and H_2_O, and the facility for scalable production, making it the first practical MOF for industrial carbon capture [26,27]. Molecular simulations suggest the CO_2_ adsorption location in CALF-20 also lies in the channel center to form interactions with the zinc/nitrogen/carbon atoms in the framework. Likewise, the amine-appended zinc-oxalate-triazolate MOFs demonstrated enhanced CO_2_ capacity at low CO_2_ concentrations compared to CALF-20, due to higher-density interaction sites and more contracted pore sizes [28,29]. Recently, we prepared a flexible MOF, namely ZnDatzBdc, that showed step-shaped CO_2_ isotherm due to breakage/reformation of intra-framework hydrogen bonds and rotation of the phenyl rings, giving rise to an excellent CO_2_ theoretical working capacity of 94.9 cm^3^/cm^3^ if performed in typical pressure vacuum swing adsorption at 273 K [30].

**Figure 1:**
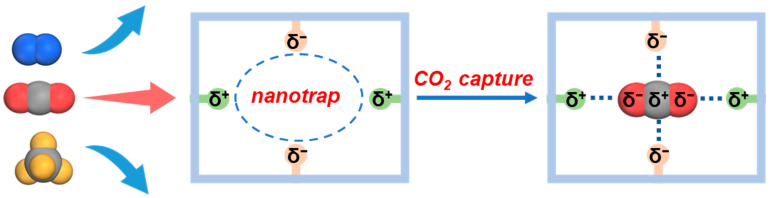
Selective CO_2_ capture from N_2_ and CH_4_ on a nanotrap with a suitable electrostatic environment via multiple host–guest interactions. The blue dotted lines represent the electrostatic interactions between the framework and the CO_2_ molecule.

By now, developing nanotraps with multiple host-guest interactions toward the target molecules offers a feasible strategy to accomplish high adsorption capacity and selectivity, which has been successfully applied in separation circumstances, such as C_2_H_2_/CO_2_ separation [31,32], C_3_H_4_/C_3_H_6_ separation [33,34], CH_4_/N_2_ separation [35], and olefin/paraffin separation [36]. For CO_2_ adsorption, if a contracted pore exhibits opposite electrostatics on the adjacent positions and the same electrostatics on its opposite side, it can form strong electrostatic interactions with both the carbon and oxygen atoms in the CO_2_ molecule, as shown in Figure 1. Hence, this type of pore can act as a suitable nanotrap for CO_2_ fixation, from which a remarkable capacity and selectivity for CO_2_ can be achieved.

Herein, we synthesized a novel MOF, namely ZnAtzCO_3_, with 3-amino-1,2,4-triazolate (Atz^-^) and carbonate (CO_3_^2−^) as dual ligands. Inspiringly, ZnAtzCO_3_ shows the desired ultramicropores due to the small-sized ligands and suitable crystal structure, which is desirable for the adsorption and separation of small molecules, such as CO_2_. Moreover, the favorable electrostatic environment of ZnAtzCO_3_ makes it a feasible nanotrap to form multiple host-guest interactions with CO_2_, and hence, efficient CO_2_ capture from N_2_ and CH_4_ could be achieved. In particular, at atmospheric temperature, equilibrium isotherms showed high CO_2_ capacities of ZnAtzCO_3_ in a wide pressure range, with values of 44.8 cm^3^/g (STP, standard temperature and pressure) at the typical fraction of the flue gas (15 kPa) and 56.0 cm^3^/g at the fraction of the biogas (50 kPa). Moreover, adsorptive selectivity based on the IAST model indicated that ultra-high CO_2_/N_2_ and CO_2_/CH_4_ selectivities of 3538 and 151 were realized at ambient conditions, respectively. The excellence in capacity and selectivity of this MOF-based nanotrap was illustrated by molecular simulations in terms of preferential adsorption sites, binding energy, and adsorption distributions. Furthermore, breakthrough experiments toward the binary mixtures of CO_2_/N_2_ and CO_2_/CH_4_ were conducted on ZnAtzCO_3_, which verified its efficient dynamic separation performance.

## 2. Results and Discussion

### 2.1. Crystal Structure and Pore Properties

**Figure 2:**
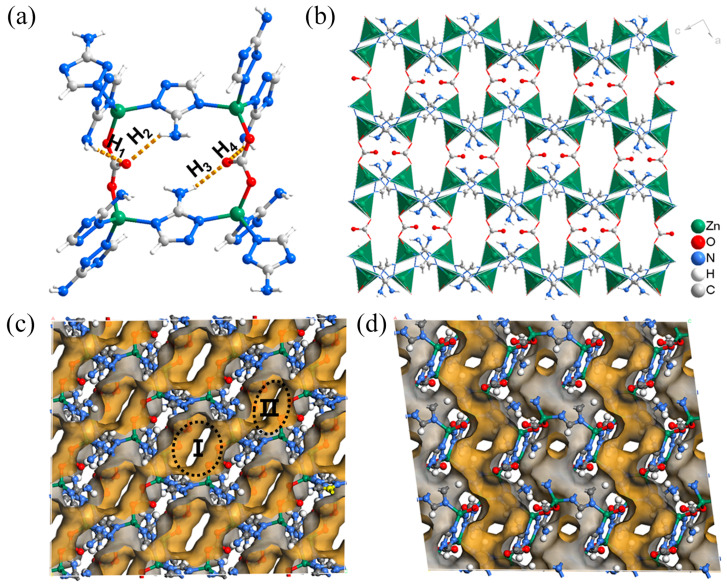
The crystal structure and pore property of ZnAtzCO_3_: (**a**) coordination mode, (**b**) crystal structure shown in the b-axis, and the Connolly surface in the b-axis (**c**) and a-axis (**d**) by using a spherical probe exhibiting a radius of 1 Å. The intraframework N-H···O hydrogen bonds are marked by the golden dotted lines. I and II in Figure 2c represent the two types of cavities on the structure.

Reactions of ZnSO_4_ and 3-amino-1*H*-1,2,4-triazole (HAtz) in a binary solution of DMF/H_2_O afforded high-quality crystals of ZnAtzCO_3_. The single-crystal X-ray diffraction (SCXRD) measurement indicates that ZnAtzCO_3_ belongs to the triclinic crystal system (a = 9.6217 Å, b = 9.6316 Å, c = 16.3408 Å, α = 81.355°, β = 86.938°, γ = 76.093°). Each asymmetric unit contains four zinc atoms, four 3-aminotriazolate ligands, and two triangular carbonate linkers (Figure S1). Because no carbonate was added to the reactants, it is assumed that the carbonate linker originated from the decomposition of the DMF molecule [37]. In addition, the elemental analysis suggests no sulfur element in the framework, which further confirms the existence of the carbonate linker instead of the sulfate or sulfite linker in the framework. As shown in Figure 2a, the zinc atom coordinates with one oxygen atom from the carbonate linker and three nitrogen atoms from three different triazolate rings. Each carbonate linker contains one uncoordinated oxygen atom, which is fixed by the intra-framework hydrogen bonding with the amino group in the triazolate ligand. We tried to obtain isoreticular structures by replacing the HAtz ligand with 1*H*-1,2,4-triazole (HTz) and 3,5-diamine-1*H*-1,2,4-triazole (HDatz), but the trial failed. Hence, we assume that these weak intra-framework hydrogen bonds are vital for the structural formation of ZnAtzCO_3_. ZnAtzCO_3_ can be regarded as a pillared–layered structure by connecting the wavy and continuous zinc-triazolate layers by the carbonate pillars (Figure 2b). Inspiringly, the small size of the carbonate and triazolate ligands gives rise to an ultramicroporous structure desirable for adsorptive separation. Specifically, ZnAtzCO_3_ contains two types of zig-zag channels with a cross-section area of 2.9 × 5.1 Å^2^ and 3.5 × 5.1 Å^2^ that interconnect with the adjacent channels through small apertures of 3.0 × 3.9 Å^2^, 2.2 × 2.8 Å^2^, and 2.6 × 3.3 Å^2^. We noticed that a similar form of Zn_2_(atz)_2_(CO_3_) was previously reported by the reaction of Zn(NO_3_)_2_, NaHCO_3_, and HAtz. Zn_2_(atz)_2_(CO_3_) displayed the same connectivity but belonged to another different space group of a Pnma unit cell (a = 9.806 Å, b = 9.3353 Å, c = 16.194 Å, α = β = γ = 90°) [38]. The difference in structure is probably because the degrees of buckling for zinc-triazolate layers can vary significantly under different synthetic conditions. Because the specific crystal structure affects the pore systems and, subsequently, the sorption behavior, the following discussion was carried out with our obtained crystal data.

### 2.2. Characterizations

**Figure 3:**
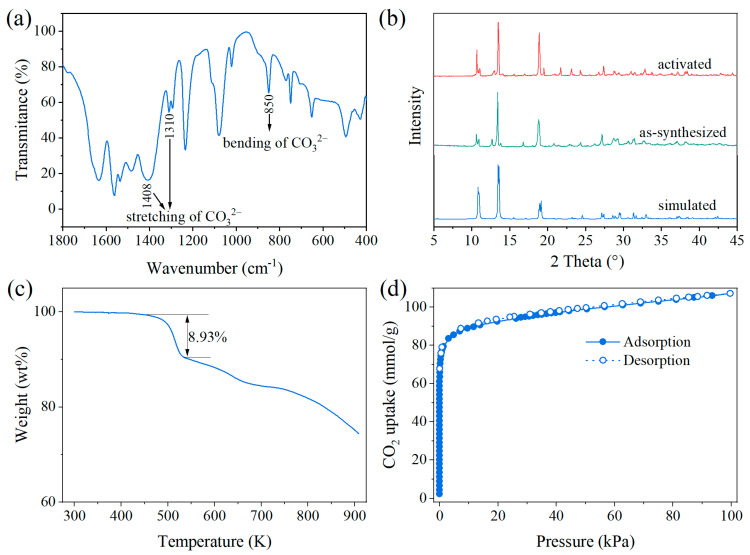
FTIR image (**a**), PXRD patterns (**b**), TG curves (**c**), CO_2_ adsorption–desorption isotherms at 195 K (**d**) of ZnAtzCO_3_.

The physicochemical behavior of ZnAtzCO_3_ was measured to investigate its textural characteristics. Fourier transform infrared reflection (FTIR) patterns were performed to further confirm the existence of the carbonate linker. Figure 3a suggests an intense broad band at 1408 cm^−1^ and an additional band at 1310 cm^−1^, corresponding to the asymmetric stretching modes of carbonate [39]. Besides, the medium band at 850 cm^−1^ was assigned to the bending mode of the carbonate. Figure 3b depicts the powder X-ray diffraction (PXRD) pattern of ZnAtzCO_3_, which is identical to that derived from SCXRD measurement, indicative of the high purity of the powder sample. In addition, the activation step did not lead to transformation or decomposition of the structure, as suggested by the well-maintained PXRD patterns. Thermogravimetric (TG) analysis in Figure 3c indicates that ZnAtzCO_3_ is stable up to 500 K, and hence, it holds enough thermal stability for adsorptive separation, which usually requires moderate heating for regeneration. The porosity feature was derived from CO_2_ sorption isotherms at 195 K, as shown in Figure 3d. The typical type-I CO_2_ isotherms indicated the microporous nature of ZnAtzCO_3_, giving a Brunauer–Emmett–Teller (BET) surface area of 455.6 m^2^/g and a micropore volume of 0.196 cm^3^/g.

### 2.3. Adsorption Equilibrium Behavior of CO_2_, N_2_, and CH_4_

**Figure 4:**
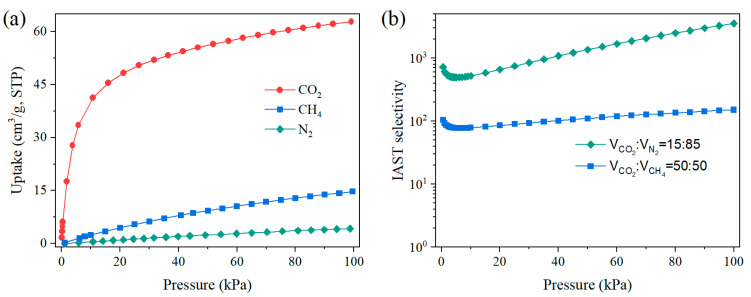
(**a**) Single-component adsorption isotherms of CO_2_, N_2_ and CH_4_ on ZnAtzCO_3_ at 298 K. (**b**) IAST selectivity of CO_2_/N_2_ (15:85, *v:v*) and CO_2_/CH_4_ (15:85, *v:v*) on ZnAtzCO_3_.

Figure 4a depicts the pure adsorption isotherms of CO_2_, N_2_, and CH_4_ on ZnAtzCO_3_ at 298 K. It is noticed that CO_2_ capacity increased sharply at low pressure, while N_2_ and CH_4_ uptakes showed a slow increment as the pressure rose. Hence, ZnAtzCO_3_ showed a significantly higher CO_2_ capacity than N_2_ and CH_4_ between 0–100 kPa. At atmospheric pressure, CO_2_ capacity reached as high as 62.8 cm^3^/g, while this value for N_2_ and CH_4_ was 4.3 and 14.7 cm^3^/g, respectively. This distinction in adsorption capacity suggests excellent thermodynamic separation for CO_2_/N_2_ and CO_2_/CH_4_. Moreover, ZnAtzCO_3_ demonstrated an exceptional CO_2_ adsorption capacity of 44.8 cm^3^/g at 15 kPa, highlighting its promising application prospects in low-concentration CO_2_ capture, such as CO_2_ elimination from the exhaust gas. Moreover, ZnAtzCO_3_ exhibits a high CO_2_ capacity of 56.0 cm^3^/g at 50 kPa, indicative of its potential in CO_2_ separation with higher CO_2_ concentrations, including CO_2_ removal from the biogas.

To evaluate the competitive separation of CO_2_/CH_4_ and CO_2_/N_2_ on ZnAtzCO_3_, the IAST selectivities were calculated by means of the IAST model, taking into account the composition of CO_2_/N_2_ (15:85, *v:v*) and CO_2_/CH_4_ (50:50,*v:v*) in the exhaust gas and biogas, respectively [40,41]. By incorporating the dual-site Langmuir–Freundlich (DSLF) parameter (Table S1) into the IAST model [42], the IAST selectivities were obtained and shown in Figure 4b. Significantly, CO_2_/CH_4_ and CO_2_/N_2_ selectivities are quite high in the whole pressure range of 0–100 kPa. At ambient pressure, the IAST selectivity for CO_2_/CH_4_ and CO_2_/N_2_ reached as high as 3538 and 151, respectively, which are comparable to those benchmark MOFs for selective CO_2_ capture through thermodynamic separation [12]. Hence, compromised of excellent CO_2_ capacity and selectivity, ZnAtzCO_3_ reveals great potential for selective CO_2_ capture.

**Figure 5:**
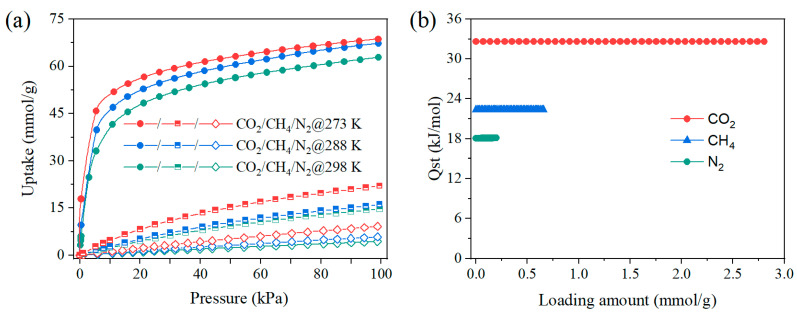
(**a**) Single-component adsorption isotherms of CO_2_, N_2_, and CH_4_ on ZnAtzCO_3_ at different temperatures (273 K, 288 K, and 298 K). (**b**) Isosteric heat of CO_2_, N_2_, and CH_4_ on ZnAtzCO_3_.

Furthermore, the isosteric heat (Q_st_) of CO_2_, N_2_, and CH_4_ on ZnAtzCO_3_, which can be derived from their pure adsorption isotherms at various temperatures (273, 288, and 298 K), is a crucial parameter for determining the adsorption interaction strengths. As shown in Figure 5b, the zero-coverage Q_st_ for the three gases followed an order of CO_2_ (32.6 kJ/mol) > CH_4_ (22.4 kJ/mol) > N_2_ (18.1 kJ/mol), consistent with the dipole moment of the guest molecules (CO_2_: 29.1 × 10^−25^ cm^3^, CH_4_: 25.9 × 10^−25^ cm^3^, N_2_: 17.4 × 10^−25^ cm^3^) [43]. Additionally, it is apparent that the Q_st_ for all gases remained constant between 0–100 kPa, suggesting homogeneity on the pore surface. From the Q_st_ result, we speculate that the remarkable CO_2_ selectivity is primarily attributed to its highest adsorption enthalpy.

In addition, ZnAtzCO_3_ was compared with other MOFs constructed by triazolate linkers on their CO_2_ uptake at 15 kPa and 100 kPa, together with the isosteric heat [25,26,29,30,44,45,46,47]. As shown in Table 1, the CO_2_ uptakes on ZnAtzCO_3_ exceed those of the ZnF(Tz) series, ZnDatzBdc, Zn(FA)(datrz)_2_, and Zn_2_(TRZ)_2_(BDC), and are comparable to that of ZU-301, and slightly lower than those of ZnAtzOx and CALF-20. Hence, ZnAtzCO_3_ holds comparatively high CO_2_ capacity among these triazolate-based MOFs. Hence, ZnAtzCO_3_ can be regarded as a promising adsorbent in combination with good capacity and selectivity toward CO_2_.

### 2.4. Molecular Simulations on the Selective CO_2_ Adsorption over N_2_ and CH_4_

**Figure 6:**
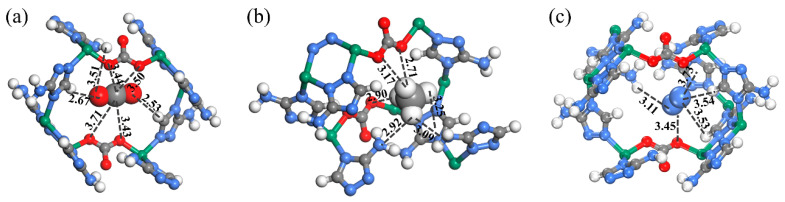
Preferential adsorption sites on the ZnAtzCO_3_ structure for CO_2_ (**a**), CH_4_ (**b**), and N_2_ (**c**) on ZnAtzCO_3_. The dashed line represents the host–guest interactions between the ZnAtzCO_3_ and the gas molecules. The unit of the distance between the gas molecules and the adsorption site is Å.

The intrinsic mechanism for the excellent separation performance on ZnAtzCO_3_ was illustrated by molecular simulations on the preferential adsorption sites, adsorption density distributions, and interaction energy with the aid of Material Studio 7.0 [48]. To visualize the intrinsic host–guest interactions between ZnAtzCO_3_ and the gas molecules, the preferential interaction site was calculated and depicted in Figure 6. Specifically, for the CO_2_ molecule, the carbon atom can form four C^δ+^···^δ−^O-C electrostatic interactions with the oxygen atoms in the carbonate linker, and each oxygen atom can form O^δ−^···^δ+^H-C electrostatic interactions with the hydrogen atoms in the aminotriazolate rings. The multiple host–guest interactions verify that the favorable electrostatic environment of ZnAtzCO_3_ can form an efficient nanotrap for CO_2_. It is noticed that the amine group did not form electrostatic interactions with CO_2_, which might originate from insufficient contact with CO_2_. For CH_4_, CH_4_ interacts with three oxygen atoms in the carbonate linker, two nitrogen atoms in the amino groups, and one adjacent triazolate ring through dispersion forces. Considering its significantly smaller polarizability and more inert nature, the preferential site displayed weaker affinity for CH_4_ than CO_2_. Likewise, being the weakest adsorbate, N_2_ forms dispersion interactions with three hydrogen atoms in the aminotriazolate linkers and one oxygen atom in the carbonate linker. From the result above, ZnAtzCO_3_ shows stronger host–guest interactions with CO_2_ compared to N_2_ and CH_4_.

**Figure 7:**
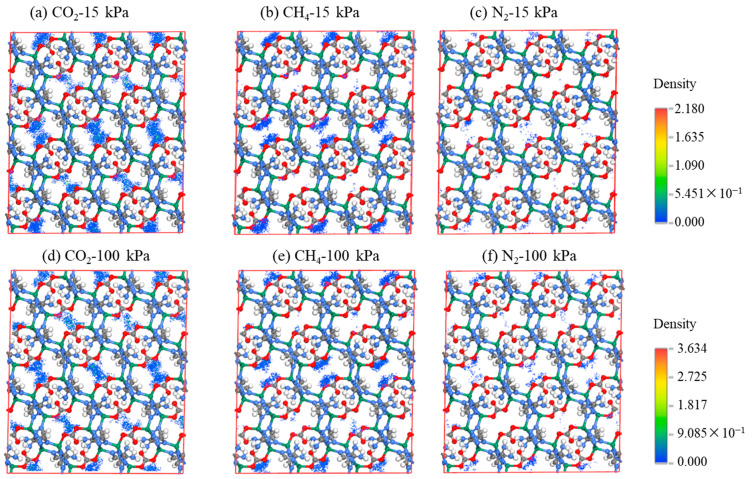
(**a**–**c**) The simulated adsorption density distribution of CO_2_, CH_4_, and N_2_ on ZnAtzCO_3_ crystal framework at 15 kPa. (**d**–**f**) The simulated adsorption density distribution of CO_2_, CH_4_ and N_2_ on ZnAtzCO_3_ crystal framework at 100 kPa.

Figure 7 presents the ambient-temperature adsorption density distributions of the three gases on ZnAtzCO_3_ at low (15 kPa) and ambient pressure (100 kPa). At both 15 kPa and 100 kPa, the density distribution for CO_2_ was the highest, followed by CH_4_ and N_2_, which is a valid proof of the significantly higher capacity of CO_2_ than N_2_ and CH_4_. As the pressure rose from 15 kPa to 100 kPa, the adsorption density increased for all gases because, generally, the increment in pressure can provide an increasing driving force for gas adsorption.

**Figure 8:**
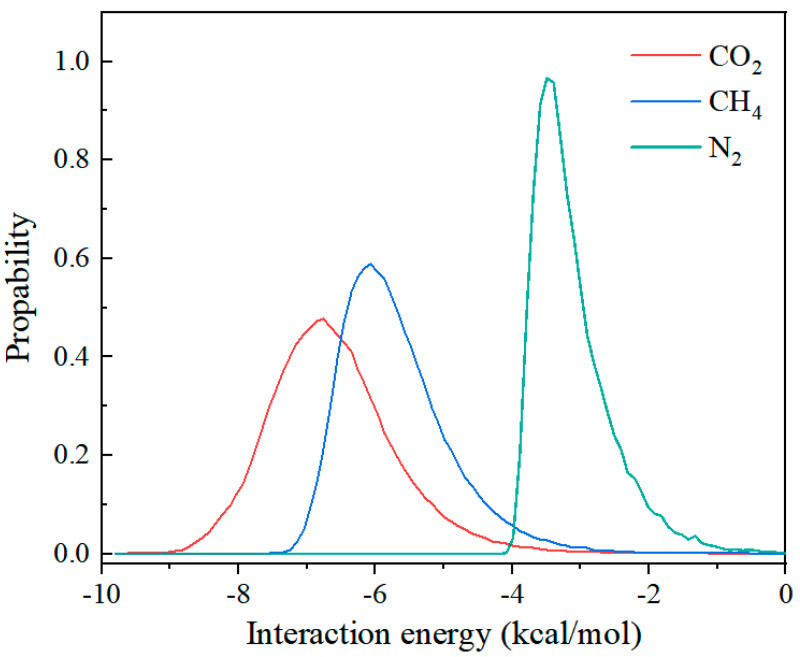
The simulated interaction energy for CO_2_, CH_4_, and N_2_ on ZnAtzCO_3_.

In addition, the distinction of the simulated interaction energy was calculated to further confirm the difference in the adsorption enthalpy. As shown in Figure 8, the average energies between ZnAtzCO_3_ and the gas molecules were −6.85, −6.06, and −3.50 kcal/mol for CO_2_, CH_4_, and N_2_, respectively, which shows a consistent trend with the Q_st_ result. Hence, the stronger electrostatic host–guest interactions, apparently higher adsorption density distributions, and larger adsorption energy, comprehensively explain the selective CO_2_ adsorption over N_2_ and CH_4_ on ZnAtzCO_3_.

### 2.5. Dynamic Breakthrough Experiments

**Figure 9:**
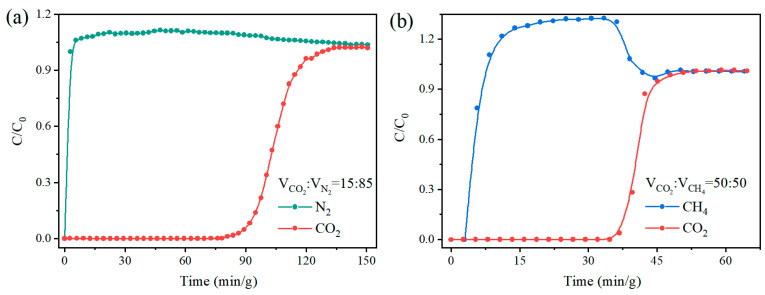
Breakthrough curves for CO_2_/N_2_ (15:85, *v:v*) (**a**) and CO_2_/CH_4_ (50:50, *v:v*) (**b**) mixture on ZnAtzCO_3_ at 298 K and 100 kPa.

For evaluation of the capability for selective CO_2_ capture from the exhaust gas and biogas on ZnAtzCO_3_, the breakthrough experiments were performed to simulate the dynamic separation performance toward CO_2_/N_2_ (15:85, *v:v*) and CO_2_/CH_4_ (50:50, *v:v*) at ambient conditions (Figure 9). It is noticed that CH_4_ and N_2_ were detected shortly after induction of the gas mixtures and reached equilibrium rapidly, confirming their uptake was inappreciable on ZnAtzCO_3_. In contrast, CO_2_ broke through at 81 min/g and reached equilibrium at 134 min/g for the CO_2_/N_2_ mixture, and the breakthrough time and equilibrium time for CO_2_/CH_4_ mixture were 37 and 53 min/g, respectively. In addition, the CO_2_ breakthrough time remained constant for five cycles for both mixtures, suggesting their excellent cyclicity in real-world CO_2_ capture conditions.

## 3. Materials and Methods

### 3.1. Material Sources

Zinc sulfate heptahydrate (ZnSO_4_·7H_2_O, 99.9%), 3-amino-1*H*-1,2,4-triazole (HAtz, 99.9%), *N*,*N*’-dimethylformamide (DMF, 99.5%), and methanol (MeOH, 99.5%) were supplied by Macklin Biochemical Co., Ltd. (Shanghai, China). The gases employed in this work, including single-component gases (CO_2_, CH_4_, N_2_, and He) of high purity (over 99.9%) and binary gas mixtures of CO_2_/N_2_ (15:85, *v:v*) and CO_2_/CH_4_ (50:50, *v:v*), were purchased from Kedi Gas Chemical Industry Co., Ltd. (Foshan, China).

### 3.2. Synthesis of ZnAtzCO_3_

HAtz (84.0 mg, 1 mmol) was dispersed in DMF (2 mL) in an autoclave, followed by addition of an aqueous solution (8 mL) of dissolved ZnSO_4_·7H_2_O (287.6 mg, 1 mmol) under stirring. After the suspension was mixed by stirring for 15 min, the autoclave was sealed, kept at 423 K for 60 h, and subsequently slowly cooled to ambient temperature. The resultant colorless crystals were recovered by filtration, washed with deionized H_2_O (2 × 20 mL) and MeOH (3 × 20 mL) to remove the unreacted reactants, and then dried in air. The activated ZnAtzCO_3_ was prepared by heating to remove the contaminants such as DMF, H_2_O, and the gases adsorbed from air. CCDC number: 2297804. Crystal Data for Zn_4_C_10_H_12_N_16_O_6_ (M = 713.92 g/mol): triclincic, space group P-1 (no. 2), a = 9.6217(2) Å, b = 9.6316(2) Å, c = 16.3408(4) Å, α = 81.355(2) °, β = 86.938(2)°, γ = 76.093(2)°, V = 1453.01(6) Å^3^, Z = 2, T = 150.00(10) K, μ(CuKα) = 0.790 mm^−1^, D_calc_ = 1.632 g/cm^3^, 5103 reflections measured (4.735° ≤ 2Θ ≤ 66.988°), 4762 unique (Rint = 0.0381, Rsigma = 0.0300) which were used in all calculations. The final R_1_ was 0.0623 (I > 2σ(I)) and wR_2_ was 0.1667 (all data). Elemental analysis (wt%) calculated for Zn_4_(Atz)_4_(CO_3_)_2_ (Zn_4_C_10_H_12_N_16_O_6_): C, 16.83; H, 1.69; N, 31.40; S: 0.00. Found: C, 17.25; H, 2.05; N, 30.5; S, 0.00.

### 3.3. Characterizations

SCXRD analysis was carried out on a Rigaku Oxford Diffraction (Rigaku, Tokyo, Japan) with a hybrid pixel array detector. Reflections combined with SHELXL corresponding to the crystal class were employed for the calculation of statistics and refinement to solve the non-hydrogen atoms, while the locations and numbers of all hydrogen atoms were calculated theoretically. Elemental analysis was performed on a Vario EL elemental analyzer (Elementar, Langenselbold, Germany) in the CHNS mode. FTIR spectroscopy in the range of 1800–400 cm^−1^ was recorded on a Thermo Scientific iN10 (Thermo Fisher Scientific, Waltham, MA, USA) microscope with potassium bromide as the matrix. PXRD patterns were collected on a Bruker D8 Advance diffractometer (Bruker, Mannheim, Germany). TG measurements of the as-synthesized ZnAtzCO_3_ were performed on a TGA 550 thermal gravimetric analyzer (Thermo Fisher Scientific, Waltham, MA, USA), and the sample was heated from 303 K to 973 K at a ramping rate of 10 K/min under flowing nitrogen.

### 3.4. Single-Component Gas Sorption Isotherm Measurements

Single-component sorption isotherm measurements between 0–100 kPa were carried out on 3Flex (Micromeritics, Norcross, GA, USA) at various temperatures. In the preparation process, approximately 100 mg of ZnAtzCO_3_ was activated under dynamic vacuum for 6 h at 393 K to afford a guest-free sample. During the test, the sample tube was placed in a thermostatic environment by using the ice-acetone bath (195 K) or circulating water bath (288 K, 298 K, and 313 K) to maintain a constant operational temperature.

### 3.5. Adsorption Selectivity Based on IAST Model

Before the IAST selectivity calculation, the experimental isotherms of CO_2_, N_2_, and CH_4_ require accurate fitting to a mathematical model. In this work, the DSLF equation, based on the assumption that two types of adsorption sites are present in the structure, was selected and described to describe the adsorption equilibrium of the single-component gases Equation (1) [30,42].

(1) $$q=q_{e1}\frac{k_{1}p^{t_{1}}}{1+k_{1}p^{t_{1}}}+q_{e2}\frac{k_{2}p^{t_{2}}}{1+k_{2}p^{t_{2}}}$$

where *p* is the specific pressure when the gas phase and adsorbed phase reach a steady state; *q_ei_* is the saturated uptake of site *i*; *k_i_* is the affinity coefficients of site *i*; *t_i_* represents the divergence from an absolute homogeneous surface on site *i*.

The IAST selectivity for CO_2_/N_2_ and CO_2_/CH_4_ on ZnAtzCO_3_ can be derived according to Equation (2) [40].

(2) $$S_{AB}=\frac{x_{A}/x_{B}}{y_{A}/y_{B}}$$

where *x_i_* and *y_i_* refer to the volume fractions of component *i* in the adsorbed phase and the gas phase, separately.

### 3.6. Isosteric Heat (Qst) Calculation

The experimental adsorption isotherms of CO_2_, N_2_, and CH_4_ on ZnAtzCO_3_ at various temperatures were fitted to the Virial equation, Equation (3) [49], and the parameters are shown in Table S2.

(3) $$\ln\left(P\right)=\ln\left(N\right)+(\frac{1}{T})\sum_{i=0}^{m}a_{i}N^{i}+\sum_{j=0}^{n}b_{j}N^{j}$$

where *p* is the pressure, *N* is the gas capacity, *T* is the absolute temperature, *a_i_* and *b_j_* refer to the corresponding parameter in the Virial equation, while *m* and *n* refer to the number required for the accurate fitting of the Virial equation.

Subsequently, the corresponding *Q_st_* was figured out by substituting the parameter in the Virial equation into the Clausius–Clapeyron equation, Equation (4) [50].

(4) $$Q_{st}=-R\sum_{i=0}^{m}a_{i}N^{i}$$

where *R* is short for the ideal gas constant, 8.314 J/mol/K.

### 3.7. Breakthrough Experiments

Dynamic separation experiments of CO_2_/N_2_ (15:85, *v:v*) and CO_2_/CH_4_ (50:50, *v:v*) mixtures were performed on self-assembly breakthrough equipment (Figure S3). A small-scale adsorption column was prepared by loading approximately 600 mg of the activated ZnAtzCO_3_ in a stainless-steel column (Φ 50 × 150 mm). For activation, the column packed with the sample was heated at 393 K for two hours to eliminate the adsorbed contaminants. Subsequently, the column was inserted into the breakthrough equipment and purged by He flow (10 mL/min) at ambient conditions until the baseline was flattened. Finally, the gas was shifted to CO_2_/N_2_ or CO_2_/CH_4_ at a flow rate of 3 mL/min. The outlet component was monitored on a thermal conductivity detector (TCD) until the outlet composition reached that of the feed gas, which suggested the breakthrough column reached equilibrium. The adsorption column was recovered by purging He flow at 373 K to liberate the adsorbed gas molecules in the cyclability test.

### 3.8. Simulation Details

The molecular simulations on the adsorption mechanism were calculated by utilizing the Materials Studio 7.0 software [50]. First, the structures of ZnAtzCO_3_ and the adsorbates were optimized with the aid of the Forcite and Dmol3 modules. The adsorption characteristics, including the optimal adsorption sites, adsorption density distribution, and stabilized adsorption energy, were simulated in the Sorption module with the Metropolis Monte Carlo method. The adsorption behavior of the guest molecules on ZnAtzCO_3_ was described by several motion types, including exchange, conformation, rotation, translation, and regeneration. The Ewald and atom-based methods were employed to depict the electrostatic interaction and Van der Waals interactions between the structure and the guest molecules, respectively. The cutoff for the Metropolis Monte Carlo simulation was set as 12.5 Å. One gas molecule was randomly inserted into the framework in the Location task in the Sorption module, with 1 × 10^5^ steps for equilibrium and production, separately.

## 4. Conclusions

In conclusion, we propose an interesting type of MOF-based nanotrap, namely ZnAtzCO_3_, for efficient selective capture of CO_2_ from N_2_ and CH_4_. The favorable electrostatic environment and narrow pore geometry of ZnAtzCO_3_ show stronger interaction with CO_2_ than N_2_ and CH_4_. Specifically, ZnAtzCO_3_ accomplished high CO_2_ capacities with values of 74.0 cm^3^/cm^3^ at the fraction of the flue gas (15 kPa) and 91.4 cm^3^/cm^3^ at the fraction of the biogas (50 kPa), together with ultra-high CO_2_/N_2_ and CO_2_/CH_4_ selectivities of 3538 and 151 at ambient conditions, respectively. This excellent separation performance was comprehensively explained by molecular simulations, which suggests that the carbon atom of CO_2_ can form strong electrostatic C^δ+^···^δ−^O-C interactions with the oxygen atoms in the carbonate ligand and the oxygen atom of CO_2_ can interact with the hydrogen atoms in the triazolate ligand through O^δ−^···^δ+^H-C interactions, enabling ZnAtzCO_3_ as an optimal nanotrap for CO_2_ fixation. Moreover, breakthrough experiments confirm excellent dynamic separation toward CO_2_/N_2_ and CO_2_/CH_4_ on ZnAtzCO_3_, highlighting its potential for selective CO_2_ capture. Furthermore, constructing suitable nanotraps with optimal electrostatic environment and pore geometry is worthy of further exploration in other separation circumstances.

## Figures and Tables

**Table 1:** Comparisons of CO_2_ uptakes at 15 kPa and 50 kPa on typical MOFs constructed by triazolate linkers.

MOFs	Q_CO2_ at 15 kPa (cm^3^/g, STP)	Q_CO2_ at 50 kPa (cm^3^/g, STP)	Q_st_ (kJ/mol)	T (K)	Ref.
MAF-7	4.5	12.5	25	298	[44]
ZnF(TZ)	6.0	19.1	24	298	[25]
ZnF(daTZ)	21.4	34.1	33	298	[25]
ZnDatzBdc	1.9	5.8	29	298	[30]
CALF-20	53.7	68.3	33.5	303	[26]
ZnAtzOx	60.5	65.0	55	303	[29]
ZU-301	47.7	52.6	39	298	[45]
Zn(FA)(datrz)_2_	12.3	28.5	24.0	298	[46]
Zn_2_(TRZ)_2_(BDC)	23.5	41.4	-	298	[47]
Zn_2_(TRZ)_2_(FA)	34.7	73.9	-	298	[47]
ZnAtzCO_3_	44.8	56.0	32.6	298	This work

## Data Availability

Data are contained within the article.

## Supplementary Materials

The following supporting information can be downloaded at: https://www.mdpi.com/article/10.3390/molecules28237908/s1, Table S1: Fitting parameters of the DSLF model for isotherms of CO_2_, N_2_, and CH_4_ on ZnAtzCO_3_; Table S2: Fitting parameters and correlation coefficients of the Virial equation for all gases on ZnAtzCO_3_; Scheme S1: Synthetic pathways for ZnAtzCO_3_ with starting reactants of ZnSO_4_/Hatz/DMF/H_2_O at 523 K; Figure S1: Asymmetric unit of ZnAtzCO_3_; Figure S2: Pore size distribution of ZnAtzCO_3_ based on the HK model using CO_2_ as the probe molecule; Figure S3: CO_2_ breakthrough times or CO_2_/N_2_ (*v:v*, 15:85) and CO_2_/CH_4_ (*v:v*, 15:85) mixtures in five consecutive cycles of breakthrough experiments on ZnAtzCO_3_.
