# Supplementary material for: Efficient Selective Capture of Carbon Dioxide from Nitrogen and Methane Using a Metal-Organic Framework-Based Nanotrap
Source: Molecules. 2023 Dec 2;28(23):7908. doi: 10.3390/molecules28237908 (PMC10708271; doi:10.3390/molecules28237908)
Supplement: Supplementary file 1 [file molecules-28-07908-s001.zip › Supporting information/supporting information final.pdf]

Supporting Information

Efficient Selective Capture of Carbon Dioxide from Nitrogen and Methane Using a Metal-Organic Framework-Based Nanotrap

Junjie Peng [1], Chengmin Fu [1], Jiqin Zhong [2], Bin Ye [3], Chongxiong Duan [4], Jing Xiao [5], and Daofei Lv [1,*]

[1] School of Environmental and Chemical Engineering, Foshan University, Foshan 528000, PR China

[2] GAC R&D Center, Guangzhou Automobile Group Co., Ltd., 668 Jinshan Road East, Panyu District, Guangzhou, Guangdong, PR China

[3] Appraisal Center for Environment and Engineering, Ministry of Ecology and Environment, Beijing 100012, PR China

[4] School of Materials Science and Hydrogen Engineering, Foshan University, Foshan 528000, PR China

[5] School of Chemistry and Chemical Engineering, South China University of Technology, Guangzhou 510640, PR China

* Correspondence: lvdaofei@fosu.edu.cn

S1. Fitting parameters of the DSLF model for isotherms of CO_2, N_2, and CH_4 on $ZnAtzCO_3$

Table S1. Fitting parameters of the DSLF model for isotherms of CO_2, N_2, and CH_4 on $ZnAtzCO_3$

Parameters	CO_2	N_2	CH_4
q_1	3.214	0.4353	1.335
b_1	0.192	0.002757	0.008264
c	0.756	1.063	0.9743
q_2	1.235	0.2935	0.3774
b_2	0.00099	0.001972	0.002858
t	0.751	1.122	1.029
R^2	0.9961	0.9993	0.9999

S2. Fitting parameters and correlation coefficients of the Virial equation for all gases on $ZnAtzCO_3$

Table S2. Fitting parameters and correlation coefficients of the Virial equation for all gases on $ZnAtzCO_3$

Gas	a_0	a_1	a_2	a_3	a_4	b_0	b_1	R^2
CO_2	-5811.5	3.283	-0.0027	3.37E-6	-1.147E-9	15.72	-0.0074	0.9980
N_2	-2174.2	-21.24	0.263	-0.00299	1.174E-5	10.03	0.045	0.9958
CH_4	-2690.9	1.643	0.0145	-8.75E-5	1.52E-7	10.52	-0.0030	0.9976

S3. Synthetic pathways for $ZnAtzCO_3$

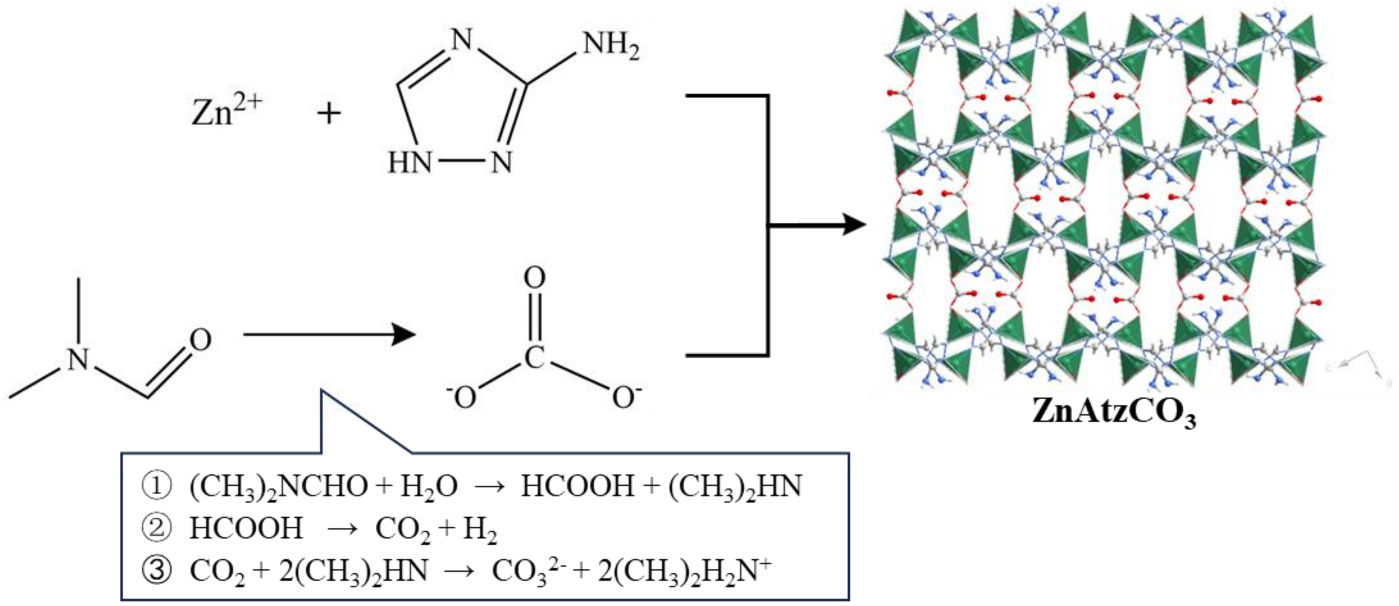

Scheme S1. Synthetic pathways for $ZnAtzCO_3$ with starting reactants of $ZnSO_4$/HAtz/DMF/H_2O at 523 K

S4. Asymmetric unit of $ZnAtzCO_3$

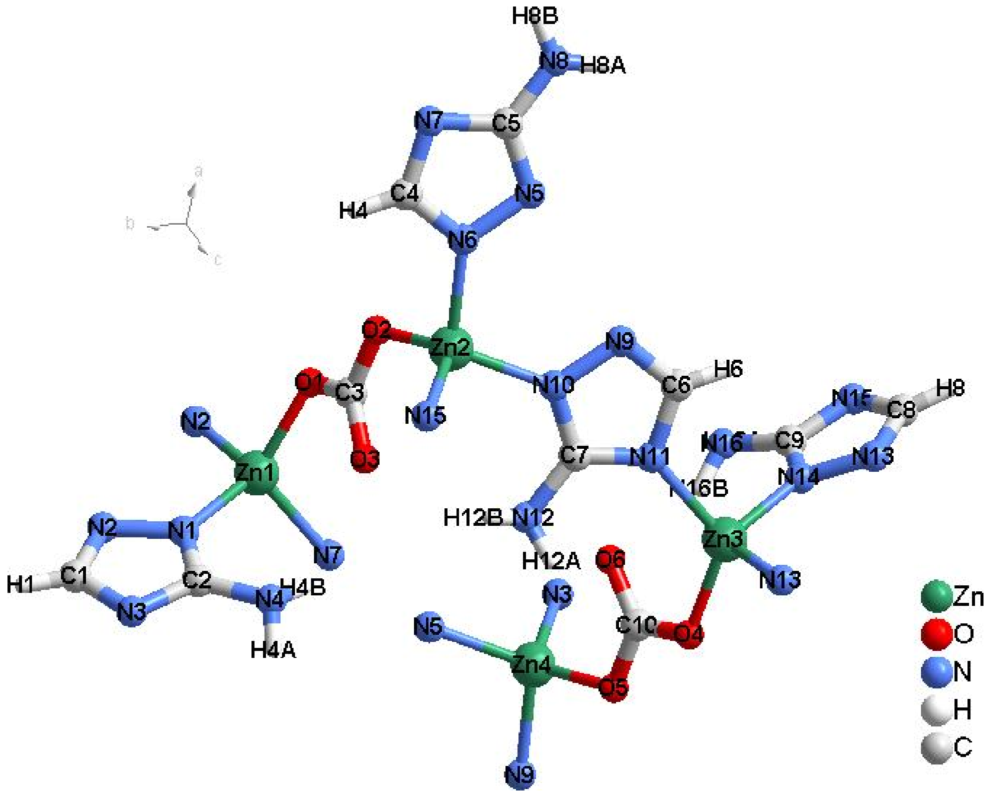

Figure S1. Asymmetric unit of $ZnAtzCO_3$

S5. Pore size distribution of $ZnAtzCO_3$ based on the HK model using CO_2 as the probe molecule

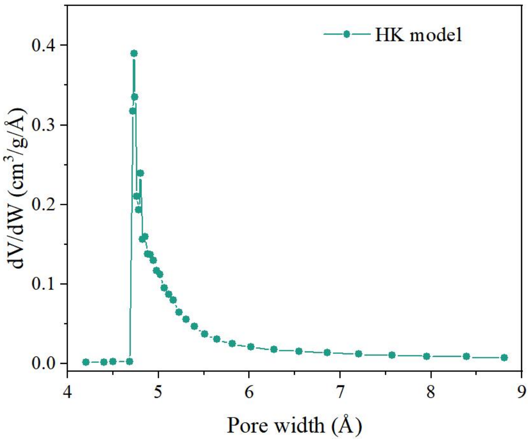

Figure S2. Pore size distribution of ZnAtzCO3 based on the HK model using CO_2 as the probe molecule

S6. CO_2 breakthrough time for CO_2/N_2 and CO_2/CH_4 mixtures in five cycles of breakthrough experiments on $ZnAtzCO_3$

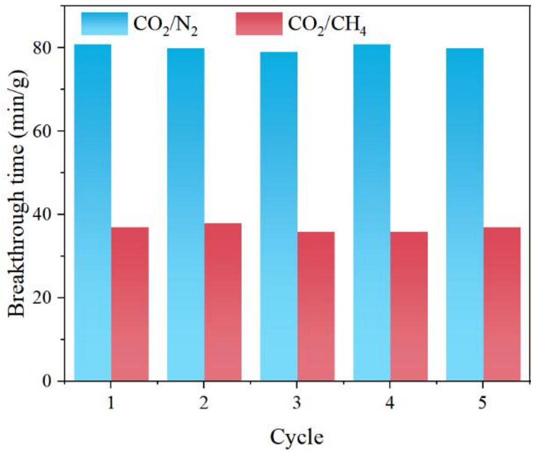

Figure S3. CO_2 breakthrough times or CO_2/N_2 (v:v, 15:85) and CO_2/CH_4 (v:v, 50:50) mixtures in five cycles of breakthrough experiments on $ZnAtzCO_3$
